# Thai Nurses’ experiences of post-operative pain assessment and its’ influence on pain management decisions

**DOI:** 10.1186/s12912-016-0136-8

**Published:** 2016-02-29

**Authors:** Manaporn Chatchumni, Ampaporn Namvongprom, Henrik Eriksson, Monir Mazaheri

**Affiliations:** School of Health, Care and Social Welfare, Mälardalen University, Västerås, Sweden; School of Nursing, Rangsit University, Pathum Thani, Thailand; Department of Nursing and Care, The Red Cross University College, Stockholm, Sweden

**Keywords:** Evidence-based practice, Pain assessment, Patient evidence paradigm, Post-operative care

## Abstract

**Background:**

While many studies have addressed various issues with regards to pain management, there is limited knowledge about how nurses assess pain in surgical wards. This study aimed to describe Thai nurses’ experiences of pain assessment in a surgical ward.

**Methods:**

A cross-sectional explorative study was conducted. Participants were selected through theoretical sampling. Data was collected through interviews with twelve registered nurses working in surgical wards. Qualitative content analysis guided the analysis of the data.

**Results:**

Nurses use a double/triple check system, communicated to the healthcare team via records and protocols, and they used their skills and experiences in pain assessment. The results showed that nurses missed the opportunity to include the patients’ self-reported pain in their accounts. Though much evidence of pain was collected, this did not seem to benefit the patients. Furthermore, the nurses were not using instruments to measure pain, which illustrates the potential unreliability of professionals who have differing opinions concerning the patients’ pain.

**Conclusions:**

Thai nurses worked based on a ‘patient-evidence’ paradigm when assessing patients in pain; this should be shifted to an evidence-based paradigm. Furthermore, by including the patients’ self-reported pain in their assessment, nurses would both improve the quality of the pain assessment and empower patients in their pain management. Pain management practices in Thailand should be improved through education, training, supportive innovation, and collegial competence development in order to improve the quality of care in the post-operative field.

## Background

Nurses play a key role in pain assessment and in advising on the standards of pain management in post-operative recovery on surgical wards. Nurses are the main providers of professional care within the post-operative care setting [1–3]. Zoëga et al. [4] reported from their study in Iceland that 57 % of the patients’ documents examined demonstrated that patients had undergone pain assessment and only 27 % had documented pain severity on a standardized scale. Pain experts agree that the widespread inadequacy of pain management has prompted efforts to improve the assessment and treatment of pain by using the most current practice guidelines of organizations, including the Agency for Health Care Policy and Research [5], the Joint Commission on Accreditation of Healthcare Organizations [6] and International Association for the Study of Pain [7].

There are many barriers that can interfere with the process of pain management. According to Shoqirat [3], many of these barriers are seen within nursing in general (e.g., staff shortages, high workloads, and the perception of 'we are nurses, they are doctors’) and more specifically in bedside nursing (e.g., attention-seeking patients, buzzer obsession, and family interferences). Insufficient reductions in patient suffering can result in increased complications and compromised hospital outcomes, such as an increase of readmission rate, a lengthy hospital stay, or higher costs of medical care [4, 8].

Previous studies conducted in Egypt and England have illustrated that higher quality pain assessment and management can be achieved with good patient observation charts and pain assessment documentation. However, these studies have also recommended that nurses should improve their knowledge and skills in pain assessment and management [9, 10]. To gain an in-depth understanding of the way nurses assess pain in surgical wards, more studies should be conducted in various contexts and settings.

Earlier studies have revealed that nurses need to improve their communication with patients, as this is necessary to determine cultural sensitivities and to provide nursing care based on the individual’s distinct values, beliefs, and traditions in a healthcare setting [4, 9]. In Thailand, pain is considered as the fifth vital sign, along with respiratory rate, temperature, heart rate, and blood pressure; the status of pain as a vital sign indicates the importance of accurately assessing patients’ pain [11]. Pain assessment is initiated by identifying patients with pain, and examining the signs of the physical illnesses or tissue injuries that bring patients to the hospital [11]. All hospitalized patients in Thailand are assessed regularly to screen for pain in order to best manage patients’ pain. In 2011, national guidelines were provided by The Royal College of Anesthesiologists of Thailand and the Thai Association for the Study of Pain demanding that nurses assess pain, regardless of patients’ expressions of pain (or lack thereof). However, the effect of these national ambitions to consider pain the fifth vital sign and routinely measure patients’ pain has actually had on the successful identification of patients in pain and the management of their pain has not yet been investigated. There may be systemic inadequacies in nursing practices in terms of under-detection and under-treatment in many patients [8, 11, 12], and current pain assessment practices in Thailand as well as other countries might be inadequate in terms of facilitating optimal pain management.

A systematic literature search for Thai articles regarding pain management, published between 1990 and 2009 [13], resulted in very few hits on how nurses assess pain in Thai contexts. One article, published by Forgeron et al. [14], addressed children’s pain assessment through the perspectives of health professionals in a Northeastern Thai context. This article focused on the under-recognition of children’s pain and the complex issues involved in communicating the findings of children’s pain; these findings might not be transferable to other health care settings. No other published articles were found that addressed pain assessment by nurses in a Thai context. The current study was designed to investigate pain assessment conducted by nurses on surgical wards. This study is part of a larger research project investigating pain management provided by Thai nurses in surgical wards following abdominal surgery. The aim of the study is to describe Thai nurses’ experiences of pain assessment in a surgical ward.

## Method

### Design

An explorative cross-sectional study with qualitative approach was conducted, which used in-depth interviews as a method of data collection [15]. Qualitative content analysis [16] guided the study to illustrate Thai nurses’ experiences of pain assessment.

### Data collection

The study was conducted in a surgical ward of a public hospital in Bangkok, Thailand; the surgical ward had a capacity of 50 beds. The selected surgical ward provided pre- and post-operative care to patients that had undergone abdominal surgery.

The data collection took place during September 2013. To recruit the potential participants, the surgical wards of the hospital were contacted and, upon acceptance by the head nurses, nurses were contacted and given information about the study. All nurses who were contacted agreed to participate in the study. Data was collected by theoretical sampling based on Benner’s nursing theory [17], and the sampling was guided by Benner’s five levels of proficiency: novice (1 to 3 years of experience), advanced beginner (4 to 5 years of experience), competency, proficiency, and expert competency (more than 10 years of experience). The study recruited a total of 12 nurses, including three registered nurses (RNs) in each of the following four levels of proficiency: novice, advanced beginner, competency and expert competency. The following inclusion criteria were used: (i) full-time work in the surgical ward, (ii) experienced in providing surgical care for at least 1 year prior to interview session, and (iii) willingness to participate in the study.

The interviews were conducted in Thai, which was the mother tongue of the interviewer and the interviewees, in a quiet room within the hospital where the participants worked. The interviews lasted approximately 45–60 min, and all interviewees were given enough time to express themselves. The interviews were recorded with a digital voice recorder, with the participants’ permission, and were later transcribed verbatim. The contact information of the interviewers was provided to the interviewees, in case they wished to share additional reflections at a later date. The first author (MC) and second author (AN) discussed the qualitative interviews in terms of sufficiency and richness of the contents [16]. Recruitment of participants continued until no new content came up in the interviews.

Prior to the interviews, a semi-structured set of interview guidelines was designed and contained the following questions:

- *How do you know when the patients are in pain?*
- *How do you measure the patients’ pain and what do you utilize in measuring the pain? And please, could you tell me how you assess pain in your daily practice?*
- *If the patient’s pain is not relieved by painkillers, how do you assess their pain afterwards?*
- *When someone has been expressing a high level of pain and/or differences exist between individuals, how do you assess their pain?*
- *Do you have a way of sharing strategies with the other team members or other nurses in the pain assessment practice in order to manage the pain of the patients?*
- *Probes for each question were as follows: Could you describe more about that? Could you give me an example of that? What does that mean to you?*

### Data analysis

The data analysis was conducted manually, according to qualitative content analysis [16]. First, the verbatim-transcribed texts were read through several times to get a sense of the whole transcript. Second, all twelve interview texts were divided and coded, considering the aim of the study. Third, context was considered in relation to specific terms or content. Then they were condensed into a description that was closely related to the texts, making it possible to interpret the underlying meaning of both the latent and manifest contents. Finally, these descriptions were condensed and abstracted into shortened sentences, which were then organized into labeled categories; these categories were further organized into themes. All the interview texts were assessed to identify similarities and differences in the text content and to establish themes.

To ensure the rigor of the study, the principles of trustworthiness in qualitative research [18] were followed. The Thai transcriptions were translated to English under close supervision of the second author (AN), who has expertise in the subject area, qualitative research and the Thai language. All the authors participated in analysis process and in developing categories.

### Ethical considerations

This study was approved by the Hospital Research Ethics Committee of Thailand (Code: 16/2555), and the Ethical Review of Research Involving Humans, Sweden (Code: 2012/383). All data were treated confidentially. The participants were fully informed of all aspects of the study and their rights, and signed a written consent.

## Results

The participants included 12 RNs who worked full time, ranging in age from 23 to 49 (median age: 38 years). Most of the nurses were female (*n* = 9) and all participants had at least a Bachelor’s degree in Nursing Sciences (see Table 1).

**Table 1:** Participants’ characteristics

Characteristics			n
Age (years old)	Range (min-max)	23–49	12
	Median	38	
Gender	Female	9	12
	Male	3	
Experience in nursing care (years)	Novice (1–3)	3	
	Advanced beginner (4-5)	3	
	Competency & expert competency (>10 years)	6	
	Range (mix-max)	1–28	
	Mean	11.33	
Education	BScN	11	
	MScN	1	
	Special education and training in pain	Yes	2
	management	No	10

The main theme that emerged was termed “*patient-evidence assessment in clinical practice”.* The categories that described the nurses’ experiences of pain assessment in a surgical ward included: (i) double/triple check system, (ii) communication via records and protocols, and (iii) using skills and experience (see Table 2). Nurses mainly collected objective evidence of patients’ pain using multiple scales as a basis for their pain management strategies. This monitoring of patients and their pain was done routinely every 4 h in the surgical ward, and included using pain scores as the fifth vital sign (i.e., temperature, respiratory rate, blood pressure, heart rate and pain scores) until they were discharged from the hospital. If the patient was asleep, the assessment was completed shortly after the patient had woken up. Each of the three categories is presented below and supported with participants’ quotations.

**Table 2:** Overview of codes, categories and a theme developed from the content analysis of pain assessment the Thai nurses in surgical wards

Theme	Patient evidence assessment in clinical practice		
Category	Double/triple check system	Communication via records and protocols	Using skills and experiences
Codes	- Using verbal scales to assess pain	- Prescriptions based on patients’ expressions	- Variations in pain assessment skills
	- Judging patients’ pain based on appearance and mobility	- Incompatibility of patients’ expressions and nurses’ documentation	- Differences in interpretation of patients in pain
	- Consulting patients’ documentation		

### Double/triple check system

The nurses described the procedure they used when assessing patients’ pain; this procedure is a double/triple check system, a multi-method approach to pain assessment involving the following: (i) using a verbal scale to assess pain, (ii) judging patients’ pain based on appearance and mobility, and (iii) consulting the patients’ documentation. The nurses and nurses’ aides used face scales and a numeric scale as tools in their routine practice. Nursing aides recorded pain scores every 4 h, and the nurses would re-assess all those patients with pains scores higher than five according to the hospital’s protocol of pain management in the hospital. Below is an excerpt of an interview with one of the participants:

*‘For me, I assess pain by looking at their facial expressions and asking about their level of pain to give it a score’* (Advanced 5)

The nurses must reassess a patients’ pain if they have a pain score higher than five (out of ten) or if the patient has requested pain medication. The nurses mentioned that it was difficult to correctly assess and manage the patients’ pain level, and to decide when to give pain medication. An example of this is seen in the following excerpt:

I always ask the patients, ’Are you in pain?’ Em…I have noticed one in three of the patients is in pain. He had told me he had mild pain level, estimated as two or three (out of ten). But he changed his habits, becoming agitated and complaining of discomfort, like colicky pain, and he became a bit aggressive. And he was moving around in his bed…he expressed it as being quite painful. I then have to judge whether to give him pain medication or not. (Beginner 2)

The nurses also judged patients’ pain based on their appearance and mobility, and investigated any potential complications by conducting physical examinations. The nurses often rechecked the pain levels in order to clarify and ensure that the recorded pain levels corresponded to the causes of the pain and suffering. Nurse’s conducted physical examinations through abdominal examination and noted any abdominal distention, the presence or absence of pain, any bleeding from the wound, whether the bladder was full and so on, in order to determine if the patients were still in pain and if their pain scores had reduced. After completing the examination, the nurses notified the physician. Below is an example excerpt describing this examination:

I have examined his wound and investigated his lower-left or right abdomen as to whether or not there was distension. Perhaps the patient had a full bladder that feels painful. I asked if the patient has any post-operative pain on the second day of recovery. Then, I observed how he walked by himself, to see if he felt pain after physical activity. (Advanced 3)

Moreover, as the following excerpt illustrates, the nurses often referred to *‘consulting the patients’ documentation’* when conducting the pain assessment, as is illustrated in the following excerpt:

I just look at the pain record as plot graphs in the graphic sheets. The physicians usually check the patients' progress as well as pain management levels by looking at the recorded vital signs on the graphic sheets… This is the information they use…to make judgments and decisions about administering pain medication. Some patients might need to decrease medication if the level of pain has been decreasing according to the graphs. (Competent 1)

The documentation was recorded six times per day (in 4-h cycles) for all patients, in accordance with the hospital’s protocol for pain management. The nurses also referred to occasionally assessing a patient’s post-operative pain by relying on his or her own experiences or by asking the relatives about the patient.

### Communication via records and protocols

This category illustrates how records and documentation of pain assessment were used for communication among the members of the team, in order to provide some continuity of care in the treatment of pain. The pain assessment process relied on routines and structure; for example, pain was evaluated and recorded as a number score every 4 h, which was plotted in the graphic sheet every 4 h and given to the physician, who then selected appropriate pain medication. It became clear that despite these protocols, this communication was particularly vulnerable to the recording of inaccurate pain levels. There was frequently an incompatibility of patients’ expressions with the nurses’ documentation, and the physicians mainly prescribed medication according to the patients’ expressions and not necessarily with regard to the nurses’ documented pain assessment.

In the excerpt below, a nurse mentions the importance of the communication document showing the graphs of pains scores and vital signs over time, as it is used by team members to manage patients’ pain:

We have plotted pain scores in the graphic sheet every 4 h. I guess that it could be an accurate representation of the patients’ pain…after giving pain medication. If the pain scores of the patients do not decrease, then I will notify the doctor or discuss with the team members as to what to do to relieve the pain of the patient. (Novice 2)

The nurses expressed as an incompatibility of the patients’ expressions and nurses’ documentation, which they felt was due to individual staff not completing the documentation records according to protocol. During the assessment certain information should be recorded, such as the morphine or pethidine dose administered, pain score, and vital signs, as well as the nurses’ notes used for recording the patient’s progress and response to treatment. Occasionally, this documentation was incomplete when the attending staff simply forgot to fill out the relevant sheets:

A patient had not told me about his pain yet, so I have to observe the patient to follow the process of completing the vital signs records. I just ask the nursing aide for the patients’ pain score, because it is their responsibility to ask the patient about their pain scores while the nurse is checking the vital signs of the patients (every 4 h). (Advanced 3)

The nurses mentioned that according to policies and guidelines, they should manage all documentation. However, the nurses had a heavy workload of caring for patients; as the documentation process was also very time-consuming, the records were sometimes not completed. The nurses mainly completed the documentation of pain assessment as a matter of routine and not with the primary intention of detecting patients’ pain and helping to alleviate it.

### Using skills and experiences

The quality of a pain assessment depends heavily on the individual nurses’ experience, as well as their level of knowledge and competence. The following two issues were found to affect the outcome of the pain assessment: (i) variation in pain assessment skills, and (ii) differences in the interpretation of patients in pain. From one nurse it became clear that the nurses' aides have a limited amount of knowledge when compared to nurses.

The nurses’ routine assessments were performed every 4 h, checking the vital signs and recording the patient’s self-reported pain score. The nurses then decided if the pain level was tolerable or required intervention; if they felt medication was needed, they would inform the physician who would then assess the patient and prescribe medication as appropriate. However, much variation was found in the nurses’ abilities to predict a patient’s pain tolerance when evaluating an individual patient’s history and their signs of pain. The level of competency in pain assessment is important, and experienced nurses generally have more comprehensive skills than novice nurses and nurses’ aides. This is important in managing pain effectively:

Perhaps I just have to look at pain scores on the vital sign recording sheet; however, these may be inconsistent with my assessment of the pain scores regarding that patients’ pain. (Novice 3)

The nurses mentioned concerns about the difference in skill levels when interpreting pain assessments; for example, considerably different levels of experience are found between the experienced nurses and the novices or nurses’ aides. Accurate pain assessments are needed for more effective pain management; the accuracy of these assessments depends heavily on the nurses’ skills and expertise in the field of post-operative care, as well as their biases and previous experiences. In practice, these differences can affect the time needed to provide pain medication to the patients. One experienced nurse described this process in the following quote:

I guess that we have too little time for sharing information, advising each patient, and providing routine care for patients in pain. Some of the staff don’t assess pain scores and also don’t reassess after administering to their patients. I am always assessing the patients’ pain scores that were inconsistent between nurse aides and me … by asking the patients questions like, ‘Do you feel pain?” Also, I spend more time conversing with the patients in order to understand and investigate their problems. I thought that is what nursing staff should do when caring for their patients… (Advanced 5)

The nurses’ abilities to measure pain varied between novice and expert nurses with regards to the accuracy of the pain assessment and their decision-making. All of the nurses expressed a well-intentioned effort to assist the patients in communicating their pain level. However, in order to prevent delay in the treatment of the patients’ pain, novice nurses remain under the supervision by an advanced beginner or competent level and/or expert nurse.

## Discussion

This study explored Thai nurses’ experiences of pain assessment in a surgical ward and showed that pain assessment carried out by the Thai nurses’ stems from what we decided to name a *“Patient evidence”* paradigm. Thai nurses’ pain assessment did not involve having the patient declare their pain level, although the patients’ self-reported pain should be considered the gold standard of pain assessment. Thai nurses tried to verify patients’ statements in various ways, including verbal numeric scales, and judging patients’ appearance and mobility. Although the patients’ direct voice and statement was the starting point of the pain assessment, the Thai nurses in this study did not include patients’ verbal expressions and complaints of pain in their assessment and documentations, but rather tried to evaluate patients’ pain through objective measurements. The nurses tended to rely heavily on routines and structures and regarded competence as an individual concern, meaning that they did not share their knowledge and experiences with one another. This approach did not foster collegial competence development, which is an important component of an evidence-based paradigm and is considered as best practice according to literature. The Thai nurses should strive to follow an evidence-based paradigm, grounded on research-based strategies, person-centered approaches, and collegial competence development.

The surgical nurses that participated in this study accumulated a lot of evidence, yet missed the point of evidence-based practice; namely, expert nurses should relay information to each other in order to make appropriate care decisions based on the information they have. The nurses often assessed pain as a routine task, during which the nurses were task-focused rather than patient-focused. Insufficient pain management is, to a large extent, due to inadequate pain assessments [9, 10, 14], and research by Mohamed, Ahamed and Mahmoud [9] has found several reasons for failures in pain assessment by nurses. Different factors appear to affect the clinical judgment of nurses, including their experience in listening, believing, and legitimizing the patient’s pain, as well as their individual skills and abilities [19, 20].

The nurses in our study did not show any considerable differences in their pain assessment with regards to their level of expertise; rather it seemed that the beginners were taught by the expert surgical nurses to follow the patient-evidence paradigm. This distinctly intuitive knowledge can been described through Benner's theoretical approach [21], in which the interpretation is based on background understanding of different situations and depends on the nurses’ confidence in trusting their colleagues’ decision-making and problem-solving skills in clinical practice. In this framework, Benner [21] states that proposed nurses ‘caring’ for different kinds of nursing approaches depended on a situation-based interpretive approach. In our study, we found that the nurses did not demonstrate this caring but rather rechecked the documentation and asked for a numeric rating of pain intensity, which had the effect of delaying nursing interventions and the treatment of pain.

Ambiguity surrounding pain assessment can further challenge nurses’ skills and competency in assessing and managing pain, as well as the effectiveness of pain management [22, 23]. Our results showed that one major problem is to act based on the patient-evidence paradigm instead of working with an *‘Evidence-based paradigm’,* which is best practice according to the literature. The nurses collected evidence through verbal numeric and face scales, and judged pain levels based on their own previous experiences of similar situation. Communication with patients and with other team members (physicians and nurses/nursing aides) did not result in the best outcome for patients. The management of the patients’ pain, whether acute or chronic, is multi-faceted because pain is presented to the nurses or physicians in many different ways [3, 20, 24]. Likewise, including the pain assessment as the fifth vital sign by the healthcare authorities did not improve the quality of pain management; this finding is in line with a previous study by Mularski et al. [25] that found that patients documented by the fifth vital sign often received insufficient pain management. In the assessment of pain, the patient is the primary person affected; pain might be detected through the patient’s symptoms in order to resolve the problem efficiently. Nurses and doctors have the responsibility to help manage and reduce the pain and suffering of their patients. However, simply relying on the schedule of pain medication prescribed by the physician lead to decreased flexibility in managing pain.

In addition, the impact of the nurses’ professional role in pain assessment seemed to be of interest in terms of the miscommunication that arises between the nurses and the patients. There are concerns about the relationship between the healthcare professionals and the patients involving the pre-existing culture and traditional contexts [26]. The nature of the patients is that they are passive recipients, in contrast to the healthcare professionals who should know best [26, 27] and there is a power relation and existing social practice with regards to an inequality between the nurses, physicians and the patients. Meanwhile, the nurses should be responsible for communication with patients to be able to understand and meet their needs and to provide appropriate care based on the pain assessment process. However, as reported in a previous study [28], the nurses only decided to be involved in the treatment of intolerable pain.

It seems as though the nurses wanted to accurately determine what the patient claims were and to record pain in other ways than just the patients’ complaints. Although a lot of evidence of pain was collected, they were not put into use for the patients’ benefit. It is important to reflect on the pain management guidelines and policy in the hospital, as this may improve staff attitudes towards sufficiency of pain management for the patient. If following an evidence-based practice paradigm, Leach [29] proposed that health professionals can still contribute the best available evidence from a situation in which decisions to deliver care are based on tradition, intuition and authority. The best practice according to Romyn et al. [30] and Whall et al. [31] for improving clinical practice is based on research-based strategies, person-centered approaches, and collegial competence development.

## Conclusions

The results of the study indicate that the participants used a patient-evidence approach to assess pain. Nurses play a pivotal role in assessing and monitoring pain, and the multi-method approach they used illustrated their nursing skills and ability to collect patient information and then rationally integrate this information with their own pre-existing beliefs, which are derived from their own experiences in pain assessment. Nursing care should follow an evidence-based paradigm that helps narrow the gaps between research and clinical practice.

Future research should further examine these pain assessment procedures, focusing on ways to improve pain assessment methods and possibly develop a set of pain management guidelines, which could ultimately improve the communication of pain assessment between the nurses and the physicians in post-operative care.

The results of this study clearly indicate that pain assessment by the Thai nurse in this study follows a patient-evidence paradigm, as opposed to the evidence-based paradigm that should become the norm. The nurses should gain their knowledge through research-based strategies, person-centered approaches and collegial competence development, and actualize improved quality of the routines and incident reports. We propose that improving the communication between the health care providers and the patients would lead to improved pain management outcomes. It would also be appropriate to empower the patients, such that they might contribute to their pain assessment with self-recorded data. Nevertheless, nurses should receive further education and training in pain assessment, and work in environments that are supportive to innovation and collegial competence; such developments would lead to improvements in the quality of care in the post-operative field.
